# Hospital Expenditure.—The Commissariat

**Published:** 1895-10-19

**Authors:** 


### The Institutional Workshop.

### HOSPITAL EXPENDITURE.—THE COMMISSARIAT.

Note.—The authorities of King's College Hospital alone refuse to supply the necessary information to enable that institution to be compared with its fellows in the present inquiry.

### III.—THE QUESTION OF NUMBERS.

In estimating the expenditure of a household, the very first step is to count heads. In reckoning the cost of hospital provisions, it has been usual to stop short at counting beds. It is of course taken for granted always that a large proportion of the expense of victualling an institution must be incurred for those whose business it is to look after the inmates proper, but the variations arising from the endless modes of administration in use are not by any means clearly understood, nor is the difference appreciated which such variations must of necessity cause in the expenses of provisions. These variations may be advantageously studied in the following table, showing the percentage of patients and of persons other than patients contained in the entire resident population of the more important London hospitals, and of some Scotch, Irish, and provincial hospitals.

London.

| | Percentage of Patients. | Percentage of Nonpatients. |
|---|---|---|
| Metropolitan | 48 | 52 |
| North-West London | 58 | 42 |
| Middlesex | 60 | 40 |
| Charing Cross | 62 | 38 |
| St. Bartholomew's | 63 | 37 |
| St. George's } | 64 | 36 |
| Royal Free } | | |
| London | 65 | 35 |
| St. Thomas's } | 66 | 34 |
| Great Northern Central } | | |
| St. Mary's } | 67 | 33 |
| Guy's } | | |
| German | 73 | 27 |
| Westminster | 75 | 25 |
| University (without nurses) | 77 | 23 |
| Seamen's | 79 | 21 |

Provincial, Scotch, and Irish.

| | Percentage of Patients. | Percentage of Nonpatients. |
|---|---|---|
| Cambridge | 50 | 50 |
| Oxford } | 60 | 40 |
| Bristol General } | | |
| Derbyshire Royal | 61 | 39 |
| Sussex County } | 64 | 36 |
| Aberdeen Royal Infirmary } | | |
| Birmingham General } | 65 | 35 |
| Dundee Royal Infirmary } | | |
| Ayr County } | | |
| Birmingham Queen's } | 67 | 33 |
| Norfolk and Norwich } | | |
| Halifax } | 69 | 31 |
| Wolverhampton } | | |
| Leeds General } | 70 | 30 |
| Newcastle Royal Infirmary } | | |
| Sheffield General } | | |
| Edinburgh Royal Infirmary } | | |
| Glasgow Royal Infirmary } | | |
| Glasgow West Infirmary } | | |
| Dublin Meath } | | |
| Bradford | 71 | 29 |
| Hull Royal Infirmary | 72 | 28 |
| York County | 73 | 27 |
| Kilmarnock | 80 | 20 |
| Belfast Royal | 82 | 18 |

To realise the full significance of these figures it must be remembered that the board of all these non-patients is divided in striking the average cost of provisions per bed among the number of patients. In the Metropolitan rather more than the cost of one official has to be borne by each bed, whereas in the Seamen's Hospital the cost of each official would be spread over nearly four beds. The result is apparent at once in the cost of provisions, if the former table be compared. The Metropolitan stands highest not only in the number of inmates, but in average for provisions among both London and provincial hospitals. Next to it stands the North-West London with again nearly one official to every patient, and a correspondingly high average per bed. And third on the London list stands the Middlesex with an unusually high average of provisions for this reason, that the hospital being closed for three months the number of patients was diminished, but the number of permanent officials, whose work was rather increased than diminished by the alterations, was not affected in anything like the same proportion. Hence the percentage of non-patients rose to forty, and the cost of housekeeping estimated per bed shows a corresponding increase.

Turning to provincial hospitals the same results may be noted. The Addenbrooke Hospital, where only 56 out of 153 beds were available, shows the high percentage of 50 persons other than patients, and a rise to £32 5s. per bed for provisions in consequence. Nothing could show more clearly than these examples of the increased cost incurred by the household during an imperative decrease in the number of patients, the want of wisdom involved in closing wards from motives of economy. In certain items savings can, of course, be effected on a reduction of patients, but it is absolutely impracticable to reduce any institution household to scale, and the inevitable result of attempting to save money by excluding some patients is to raise the cost of all the others.

So far we have considered only the hospitals overweighted with officials. At the other end of the scale come those which are exceptionally free from inmates other than patients. Among the larger London hospitals, the three showing the lowest average for provisions are University, where the nurses are not boarded by the hospital; Westminster, and the Seamen's, all three showing a correspondingly low proportion of officials boarded. Those provincial hospitals only can be indicated from which returns have been received, and here the Belfast Royal Hospital stands lowest, the number of non-patients boarded (again without including nurses) amounting to but 18 per cent of the total inmates.

It will be clearly seen if the figures are studied attentively and compared with the tables showing cost of provisions, that the reckoning of heads, although a valuable assistance in judging fluctuations of cost, is only one of many factors which determine expenditure. It has been shown to have an important share in producing extremes. But it would be idle to suppose that uniformity of numbers can produce uniformity of cost, and it may be noted that among seven hospitals showing an identical proportion of 30 per cent. of officials, and hence an identical rate of provisioning per bed, no two show precisely the same average cost of provisions.

It will be necessary to look more closely into the elements which go to make up the large proportion of "persons other than patients" boarded in the hospitals.

